# Rural-Urban Differences in Availability of Telemedicine Services Among Medicare Beneficiaries During COVID-19

**DOI:** 10.1093/geroni/igab046.1802

**Published:** 2021-12-17

**Authors:** Yvonne Jonk, Erika Ziller, Heidi O'Connor

**Affiliations:** 1 University of Southern Maine, Muskie School, Portland, Maine, United States; 2 University of Southern Maine, Portland, Maine, United States

## Abstract

The COVID-19 pandemic has created substantial disruptions to all aspects of rural and urban U.S. life. At the same time, it has provided opportunities for shifts in health service delivery, including policy innovations to increase telehealth availability and use for diagnosis and treatment of health concerns. However, it is unclear whether rural residents, particularly older adults, have the same access to telehealth services as their urban counterparts. Rural providers may face unique barriers to delivering telehealth services, and rural patients may have more difficulty accessing those services from their homes. This study used the Fall and Summer 2020 Medicare Current Beneficiary Survey COVID-19 Supplement Public Use Files to examine rural-urban differences in the telemedicine services available to Medicare beneficiaries from their primary care providers, as well as their ability to access those services. Preliminary findings suggest that rural beneficiaries are less likely to have access to telehealth services during the pandemic, they were more likely to exhibit hesitancy towards receiving the COVID-19 vaccine, they were less likely to engage in preventive behaviors such as hand washing and sterilizing surfaces, and more likely to have missed diagnostic or medical screening tests (37%) compared to urban (27%) beneficiaries. Finally, rural beneficiaries were less likely to have a smartphone, computer, or tablet at home and less likely to have access to the internet (78% rural; 84% urban). Policy implications include the need for outreach efforts to better inform the provider community, and efforts to improve rural health system infrastructure available to support telehealth.

